# Supplementary material for: Elementary sensory-motor transformations underlying olfactory navigation in walking fruit-flies
Source: eLife. 2018 Aug 21;7:e37815. doi: 10.7554/eLife.37815 (PMC6103744; doi:10.7554/eLife.37815)
Supplement: Transparent reporting form [file elife-37815-transrepform.docx]

***eLife’s* transparent reporting form**

We encourage authors to provide detailed information *within their submission* to facilitate the interpretation and replication of experiments. Authors can upload supporting documentation to indicate the use of appropriate reporting guidelines for health-related research (see [EQUATOR Network](http://www.equator-network.org/%2520)), life science research (see the [BioSharing Information Resource](https://biosharing.org/)), or the [ARRIVE guidelines](http://www.plosbiology.org/article/info:doi/10.1371/journal.pbio.1000412) for reporting work involving animal research. Where applicable, authors should refer to any relevant reporting standards documents in this form.

If you have any questions, please consult our Journal Policies and/or contact us: [editorial@elifesciences.org](mailto:editorial@elifesciences.org).

**Sample-size estimation**

- You should state whether an appropriate sample size was computed when the study was being designed
- You should state the statistical method of sample size computation and any required assumptions
- If no explicit power analysis was used, you should describe how you decided what sample (replicate) size (number) to use

Please outline where this information can be found within the submission (e.g., sections or figure legends), or explain why this information doesn’t apply to your submission:

**We did not use an explicit analysis to estimate an appropriate sample size. In other behavioral studies with Drosophila and other insects, sample sizes in the order of few tens of animals are normally used. In this aspect, almost all of our samples are larger than what is usually encountered in other studies of similar scope (75 flies for Fig. 1, n=13-15 flies for Fig. 2, n=30-40 flies for Fig. 3) We observed that a sample size of 10 flies was in most cases enough to identify the fundamental behaviors we described. We went beyond that number when we wanted to be able to observe inter-individual variability in responses (e.g. Figure 1), or when a detailed quantitative analysis was performed (e.g. Figure 3) and therefore cleaner average responses were desirable.**

**Replicates**

- You should report how often each experiment was performed
- You should include a definition of biological versus technical replication
- The data obtained should be provided and sufficient information should be provided to indicate the number of independent biological and/or technical replicates
- If you encountered any outliers, you should describe how these were handled
- Criteria for exclusion/inclusion of data should be clearly stated
- High-throughput sequence data should be uploaded before submission, with a private link for reviewers provided (these are available from both GEO and ArrayExpress)

Please outline where this information can be found within the submission (e.g., sections or figure legends), or explain why this information doesn’t apply to your submission:

**The number of flies run for each experiment is given in the legend for that figure. Section 5.4 of the Methods describes the number of trials per fly and steps taken to ensure reliability, collecting data on non-consecutive days and over several month. Data selection and processing methods are outlined in Methods section 5.5**

**S**tat**istical reporting**

- Statistical analysis methods should be described and justified
- Raw data should be presented in figures whenever informative to do so (typically when N per group is less than 10)
- For each experiment, you should identify the statistical tests used, exact values of N, definitions of center, methods of multiple test correction, and dispersion and precision measures (e.g., mean, median, SD, SEM, confidence intervals; and, for the major substantive results, a measure of effect size (e.g., Pearson's r, Cohen's d)
- Report exact p-values wherever possible alongside the summary statistics and 95% confidence intervals. These should be reported for all key questions and not only when the p-value is less than 0.05.

Please outline where this information can be found within the submission (e.g., sections or figure legends), or explain why this information doesn’t apply to your submission:

**All statistical analyses performed and sample sizes are detailed in the corresponding figure legends. Exact p-values are provided in all instances, either in the figure legend (Figure 2) or in tables in Methods section 5.6. Also, details on resampling and the corresponding estimation of standard error, together with estimation of error for binary data sets (Figure 6I) are provided in Methods sections 5.5 and 5.6.**

(For large datasets, or papers with a very large number of statistical tests, you may upload a single table file with tests, Ns, etc., with reference to sections in the manuscript.)

**Group allocation**

- Indicate how samples were allocated into experimental groups (in the case of clinical studies, please specify allocation to treatment method); if randomization was used, please also state if restricted randomization was applied
- Indicate if masking was used during group allocation, data collection and/or data analysis

Please outline where this information can be found within the submission (e.g., sections or figure legends), or explain why this information doesn’t apply to your submission:

**We did not perform any group allocation, or masking as part of this study.**

**Additional data files (“source data”)**

- We encourage you to upload relevant additional data files, such as numerical data that are represented as a graph in a figure, or as a summary table
- Where provided, these should be in the most useful format, and they can be uploaded as “Source data” files linked to a main figure or table
- Include model definition files including the full list of parameters used
- Include code used for data analysis (e.g., R, MatLab)
- Avoid stating that data files are “available upon request”

**Model parameters are provided in Tables 1 and 2. All data and analysis code will be deposited to appropriate databases upon publication.**

Please indicate the figures or tables for which source data files have been provided:
